# High HIV risk and syndemic burden regardless of referral source among MSM screening for a PrEP demonstration project in Toronto, Canada

**DOI:** 10.1186/s12889-018-5180-8

**Published:** 2018-02-27

**Authors:** James Wilton, Syed W. Noor, Alexandre Schnubb, James Lawless, Trevor A. Hart, Troy Grennan, Shawn Fowler, John Maxwell, Darrell H. S. Tan

**Affiliations:** 1 Applied Epidemiology Unit, Ontario HIV Treatment Network, Toronto, Ontario Canada; 2 Department of Psychology, Ryerson University, Toronto, Ontario Canada; 3 Division of Infectious Diseases, St. Michael’s Hospital, 30 Bond St, 4CC – Room 4-179, Toronto, ON M5B 1W8 Canada; 4 Dalla Lana School of Public Health, University of Toronto, Toronto, Ontario Canada; 5 British Columbia Centre for Disease Control, Vancouver, BC Canada; 6 Department of Medicine, University of British Columbia, Vancouver, BC Canada; 7 Hassle Free Clinic, Toronto, Ontario Canada; 8 ACT (AIDS Committee of Toronto), Toronto, Ontario Canada; 9 Department of Medicine, University of Toronto, Toronto, Ontario Canada

**Keywords:** PrEP, Gay men and other men who have sex with men, Syndemics, HIV risk, Screening

## Abstract

**Background:**

To maximize public health impact and cost-effectiveness, HIV pre-exposure prophylaxis (PrEP) must reach individuals at high HIV risk. Referrals for PrEP can be self- or provider-initiated, but there are several challenges to both. We assessed whether HIV risk differed by referral source among gay, bisexual and other men who have sex (gbMSM) screening for an HIV PrEP demonstration project.

**Methods:**

PREPARATORY-5 was an open-label PrEP demonstration project enrolling gbMSM at high risk of HIV acquisition in Toronto, Canada. Study eligibility criteria related to high risk was defined as scoring ≥10 on the HIV Incidence Risk Index for MSM (HIRI-MSM) and engaging in at least 1 act of condomless receptive anal sex within the past 6 months. Recruitment was promoted through self-referrals (ads in a sexual networking app and gay newspaper/website) and provider-referrals (10 community-based organizations, CBOs). HIV risk score (HIRI-MSM) and syndemic health burden were measured among gbMSM screened for study participation and compared according to referral source.

**Results:**

Between October 16 and December 30, 2014, online ads generated 1518 click-throughs and CBOs referred 115 individuals. Overall, 165 men inquired about the trial, of which 86 underwent screening. The majority of screened men were self-referrals (60.5%), scored ≥10 on HIRI-MSM (96.5%), and reported condomless receptive anal sex in the past 6 months (74.2%). Self- and provider-referrals had similarly high HIV risk profiles, with a median (IQR) HIRI-MSM score of 26.0 (19.0–32.5) and 28.5 (20.0–34.0) (*p* = 0.3), and 75.0% and 73.5% reporting condomless receptive anal sex (*p* = 0.9), respectively. The overall burden of syndemic health problems was also high, with approximately one-third overall identified as having depressive symptoms (39.5%), alcohol-related problems (39.5%), multiple drug use (31.4%), or sexual compulsivity (31.4%). There were no significant differences in syndemic health problems by referral source.

**Conclusions:**

HIV risk and syndemic burden were high among gbMSM presenting for this PrEP demonstration project regardless of referral source. Self-referral may be a useful and efficient strategy for identifying individuals suitable for PrEP use. Online strategies and CBOs working in gay men’s health may play important roles in connecting individuals at high HIV risk to PrEP services.

**Trial registration:**

ClinicalTrials.gov NCT02149888. Registered May 12th 2014.

## Background

Daily oral tenofovir/emtricitabine as pre-exposure prophylaxis (PrEP) is highly effective at preventing HIV among men who have sex with men when used as directed [1, 2], and was approved by the U.S. Food and Drug Administration in 2012 and Health Canada in 2016. Guidelines from the World Health Organization [3], U.S. Centers for Disease Control and Prevention [4], and others [5], recommend restricting PrEP to those at high risk of HIV infection in order to maximize public health benefits and cost-effectiveness. As implementation proceeds, a major challenge lies in identifying such individuals and referring them to PrEP services.

PrEP referrals can be either self- or provider- initiated, but each strategy has potential challenges. Providers may have trouble recognizing or assessing a client’s risk of HIV infection, in part because many HIV risk behaviours are stigmatized and therefore not discussed [6], leading to missed opportunities for referral. Indeed, many gay, bisexual and other men who have sex with men (gbMSM) do not feel comfortable discussing their sexual health with providers [7, 8], and even when HIV risk is discussed it can be difficult to assess due to the wide range of biological, behavioural, and social factors involved. Clinical HIV risk scoring tools, such as the HIV Incidence Risk Index for Men who have Sex with Men (HIRI-MSM) [9], have been developed to facilitate discussions about HIV risk behaviours and assist providers in identifying those at highest risk, but it is unclear to what extent these tools are used.

On the other hand, at-risk individuals may seek out PrEP themselves (self-refer), but we and others [7, 10, 11] have previously identified low perceived HIV risk among objectively high risk gay men (as determined by HIRI-MSM score [7], having undiagnosed HIV infection [10], or meeting the entry criteria for the iPrEX PrEP clinical trial [11]) as a potential barrier to such referrals. Possible reasons for the discordance between objective and perceived HIV risk include lack of knowledge, health-related optimism, and denial/avoidance (due to fear and shame created by stigma/discrimination) [12, 13]. Conversely, some gbMSM who self-refer may be at low risk for HIV but highly anxious about HIV transmission, or be individuals who want to use PrEP as an alternative to their pre-existing consistent condom use. PrEP use in such individuals may sometimes be clinically justifiable, but widespread uptake in these groups could undermine the cost-effectiveness of PrEP [14]. This is because more of such individuals would need to be using PrEP in order to avert a single HIV infection (ie. higher number needed to treat), thus incurring greater financial costs per infection prevented.

To explore the effectiveness of different referral strategies in directing high risk gbMSM to PrEP in Toronto, Canada, we compared the level of HIV risk among gbMSM screened for a pilot PrEP demonstration project according to whether they were self- or provider-referred. To measure HIV risk as comprehensively as possible, we used both a sexual behaviour-based index of HIV risk in gbMSM (the HIRI-MSM screening tool) and validated scales that screen for mental health problems. We compared the burden of co-occurring mental health problems in these groups because problems such as depression and substance use are tightly associated with HIV risk among gbMSM [15, 16], and because linking PrEP patients to care for such “syndemic” problems may reduce underlying HIV risk and facilitate PrEP adherence. Further, the HIRI-MSM only includes items on sexual and drug-taking behaviors, and does not include items for mental health problems and other more distal HIV risk factors, despite their objective connection to HIV risk. We have previously observed a high prevalence of syndemic problems among gay men seeking both PrEP [17] and nPEP (non-occupational post-exposure prophylaxis) [18], but these analyses were not stratified by referral type. In this study, we hypothesized that provider-referred men would have a higher HIV risk profile (as determined by their HIRI-MSM score and number of syndemic problems) than men who self-referred.

## Methods

### Trial and eligibility criteria

PREPARATORY-5 was an open-label, 12-month demonstration project at an academic hospital-based HIV clinic in downtown Toronto whose main objectives were to obtain pilot data on PrEP acceptability and clinical outcomes among Toronto gbMSM (NCT02149888). To be eligible, gbMSM had to be 18 years or older, living in the greater Toronto area, HIV-negative, and at high risk for HIV acquisition (defined as scoring ≥10 on the HIRI-MSM [9] and engaging in at least 1 act of condomless receptive anal sex within the past 6 months). The target sample size for the pilot project was 50.

### Recruitment

Participants were recruited through both self- and provider-referrals between October 16 and December 30, 2014. Self-referrals were generated through 1) advertisements on the gay social/sexual networking application *Grindr* from October 16 to 23, 2014, 2) advertisements on the LGBT newspaper/website *Xtra* from October 16 to December 30, 2014, and 3) word of mouth generated by these advertisements. To solicit provider referrals, two research team members visited 10 Toronto-area community-based organizations (CBOs) working in gay men’s health, including a major sexual health clinic popular with the city’s gay community (Hassle Free Clinic), from mid-October to mid-November, 2014. These visits provided basic PrEP information, an overview of study design, and instructions on how to refer clients to the study. Each CBO was asked to distribute uniquely numbered referral cards to clients whom they thought would benefit from PrEP. The referral cards contained study and referral information and were used to quantify number of CBO referrals. Importantly, none of the recruitment materials (advertisements, CBO visits, referral cards) explicitly described the study eligibility criteria related to HIV risk, but simply called for gbMSM at high risk of HIV infection. Interested individuals contacted a single trained research coordinator by telephone and the coordinator assessed men’s eligibility related to age and location only. Those meeting age/location criteria were offered an in-person screening visit.

### Data collection

Men who attended the screening visit and consented to study participation completed a self-administered electronic questionnaire covering sociodemographics, method of referral, reasons for interest in PrEP, and sexual behaviours. The questionnaire also included validated scales to measure HIV risk, syndemic health problems and attitudes towards sexual identity/behaviours, as detailed below.

HIV risk was quantified using the 7-item HIRI-MSM screening tool [9]. This tool was derived from two cohorts of gay men in the United States conducted in the late 1990s. Scores range from 0 to 47 based on questions related to age, number of male sex partners, number of condomless receptive anal sex acts, number of HIV-positive male partners, number of condomless insertive anal sex acts with HIV-positive partners, use of methamphetamines, and use of amyl nitrates (“poppers”). All questions ask about behaviours in the past 6 months. A score ≥ 10 is the suggested cutoff for identifying men at higher risk of HIV infection.

We used the Center for Epidemiological Studies - Depression (CES-D) scale [19] to screen for depressive symptoms. Scores range from 0 to 60 and, based on prior studies, a score ≥ 23 was considered a positive screen for a high likelihood of current major depressive disorder [20, 21]. Cronbach’s α for the CES-D scale in our sample was 0.83.

We used the 10-item Alcohol Use Disorders Identification Test (AUDIT) to screen for excessive drinking (α = 0.81) [22]. A score ≥ 8 is a recommended indicator of harmful alcohol use and possible alcohol dependence [23]. We defined multiple substance use as the use ≥2 recreational drugs (methamphetamine, cocaine, crack, ketamine, ecstasy, MDMA, GHB or poppers) in the last 3 months.

We used the Sexual Compulsivity Scale (SCS) to screen for sexual compulsivity. We defined scores ≥24 (α = 0.92) as indicating sexual compulsivity, consistent with published literature on this construct among MSM [24–26].

We then calculated syndemic count scores [15] by assigning one point each for the presence of depressive symptoms, harmful alcohol use, multiple substance use or sexual compulsivity, producing values ranging from 0 to 4.

### Statistical analysis

The primary objective was to compare our outcomes of interest (HIRI-MSM and syndemic count score) by referral source. Participants referred to the study by a CBO or an independent physician were classified as provider-referred, while those whose referral source was an advertisement, online/social media, or friend/word of mouth were classified as self-referred.

We used Pearson’s chi-square tests (for categorical variables) and Wilcoxon rank sum tests (for continuous variables) to examine whether participant characteristics differed between the two referral groups. In addition, we fit univariable and multivariable regression models with sandwich estimators to explore the association between referral source (independent variable with self-referral as referent category) and each of our outcomes of interest and their composites (dependent variables). We used linear regression for continuous outcomes, Poisson regression for count outcomes and logistic regression for binomial outcomes. For multivariable models, we controlled for race/ethnicity, education and age. Age was excluded from models with HIRI-MSM as the dependent variable of interest, as age is a composite component of this scale. For binomial models we used the *exlogistic* command in Stata (StataCorp., 2013) to fit an exact logistic regression model, which produces more accurate inferences in small samples than the standard maximum-likelihood-based logistic regression estimator. Finally, to assess whether the relationship between HIV risk and referral source varied according to the burden of syndemic conditions, we conducted a post-hoc analysis in which an interaction term between HIRI-MSM score and syndemic score was added to a multivariable model with referral source as the dependent variable. All statistical tests were two-tailed and all analyses were conducted in Stata 13.1 (StataCorp., 2013).

### Sample size considerations

To accrue the target sample size of 50 participants for the PREPARATORY-5 pilot trial, we anticipated screening 80–100 individuals, with a ratio of self-referrals to provider referrals between 1 and 1.5. In prior studies among Toronto MSM from our group, the standard deviation of HIRI-MSM scores was 8.6 points [7]. We estimated [27] that our analysis would be able to detect a true difference of roughly 5 points in the mean HIRI-MSM scores between self- and provider-referred participants, setting α = 0.05 and power at 80%. This is a clinically meaningful difference in HIRI-MSM scores, as it is similar in magnitude to the points assigned for many risk factors on the HIRI-MSM tool (eg. 5 points for methamphetamine use, 4 points per increment in number of HIV-positive sex partners).

## Results

**Fig. 1:**
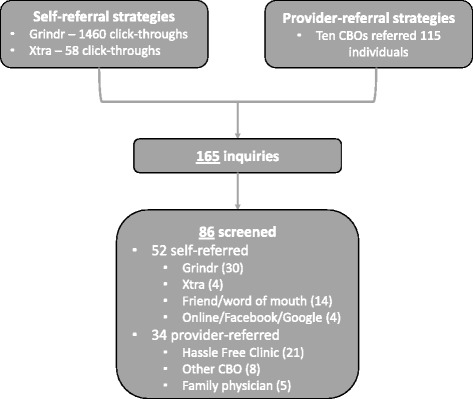
Recruitment and referral pathway to the PREPARATORY-5 demonstration project. Participants could be referred through either self or provider-referred pathways. Specific information on referral source only collected for screened participants. Number of uniquely numbered referrals cards distributed by CBOs to clients used to quantify CBO referrals. CBO = community-based organization

During the recruitment period, the advertisements on *Grindr* and *Xtra* generated 1460 and 58 click-throughs, respectively, while the 10 CBOs referred 115 individuals to the study. Overall, 165 men inquired about the trial, of which 86 underwent screening, were deemed eligible, and consented to study participation (Fig. 1).

**Fig. 2:**
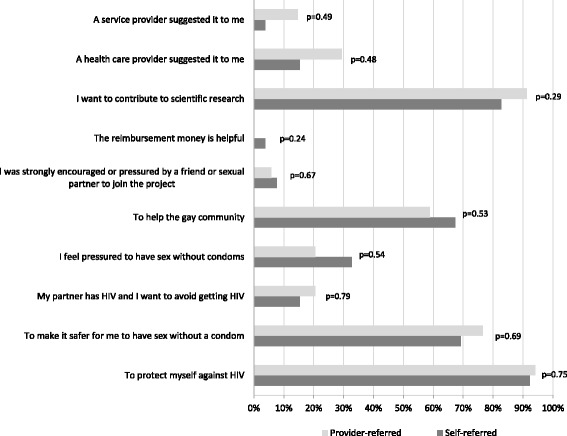
Reasons for wanting to participate in PREPARATORY-5 by referral source. Participants could provide multiple reasons for study participation. Participants were defined as self- or provider-referred based on their primary reported referral pathway, such that some self-referred participants still reported “provider suggestion” among their reasons for wanting to join the study. *P* values calculated using chi-squared analyses

The median (IQR) age of screened participants was 33 (27–40) years. The majority were White/non-Hispanic (72.1%), had a college degree or higher (76.7%), identified as gay (94.2%), and had a primary physician (80.2%) (Table 1). Prior knowledge of PrEP was very high (91.9%) and 14.0% had previously used nPEP. The majority (60.5%) were self-referrals, primarily through *Grindr*, with the remainder (39.5%) referred by providers, primarily a sexual health clinic. A quarter (26.9%) of self-referred men were referred through friends or word of mouth. The most common reasons for study participation were “To protect myself from HIV” (93.0%), “I want to contribute to scientific research” (86.1%), and “To make it safer for me to have sex without a condom” (72.1%). There were no significant differences in demographic characteristics (Table 1) or reasons for study participation (Fig. 2) by referral source.

**Table 1:** Characteristics of PREPARATORY-5 participants and comparison by referral source

	Total (*N* = 86)	Self-referred (*N* = 52)	Provider-referred (*N* = 34)	*p* ^a^
Referral Source, *n* (%)				NA
An advertisement on Xtra	4 (4.65)	4 (7.69)		
An advertisement on Grindr	30 (34.88)	30 (57.69)		
Facebook/Online/Google	4 (4.65)	4 (7.69)		
Friend/Word of mouth	14 (16.27)	14 (26.92)		
Hassle Free Clinic	21 (24.42)		21 (61.76)	
Other community based organization	8 (9.30)		8 (23.53)	Community based organization
Family physician	5 (5.81)		5 (14.71)	
Age, in years, Median (IQR)	33 (27–40)	32 (27–37)	34.5 (28–42)	0.25
Race/ethnicity, *n* (%)				0.47
White, non-Hispanic	62 (72.09)	39 (75.0)	23 (67.65)	
Non-White^b^	24 (27.91)	13 (25.0)	11 (32.35)	
Income, *n* (%)				0.08
Under $20,000	16 (18.60)	7 (13.46)	9 (26.47)	
$20,000–$39,999	19 (22.09)	11 (21.15)	8 (23.53)	
$40,000–$59,999	21 (24.42)	16 (30.77)	5 (14.71)	
$60,000–$79,999	14 (16.28)	10 (19.23)	4 (11.76)	
$80,000–$99,999	6 (6.98)	1 (1.92)	5 (14.71)	
Over $100,000	10 (11.63)	7 (13.46)	3 (8.82)	
Education, *n* (%)				0.79
No college degree	20 (23.26)	13 (25.00)	7 (20.59)	
College degree or higher	66 (76.74)	39 (75.00)	27 (79.41)	
Sexual Orientation, *n* (%)				0.99
Gay	81 (94.19)	49 (94.23)	32 (94.12)	
Bisexual	5 (5.81)	3 (5.77)	2 (5.88)	
Has a primary care physician, *n* (%)	69 (80.23)	44 (84.62)	25 (73.53)	0.21
Ever heard about PrEP, *n* (%)	79 (91.86)	48 (92.31)	31 (91.18)	0.99
Ever used PrEP, *n* (%)	12 (13.95)	5 (9.62)	7 (20.59)	0.21
Used sexual performance-enhancing drugs, last 3 months, *n* (%)	31 (36.05)	16 (30.77)	15 (44.12)	0.20
Perceived risk (0–100%) of becoming infected with HIV in next year, Median (IQR)	37.5 (20–60)	30 (20–52.5)	50 (20–60)	0.36
HIRI-MSM and component variables				
HIRI-MSM Score, Median (IQR)	26 (19–33)	26 (19–32.5)	28.5 (20–34)	0.28
Used poppers, last 3 months, *n* (%)	52 (60.47)	26 (50.00)	26 (76.47)	0.01
Used amphetamine, last 3 months, *n* (%)	12 (13.95)	8 (15.38)	4 (11.76)	0.76
Age				
< 18 years, *n* (%)	0 (0.0)	0 (0.0)	0 (0.0)	0.20
18–28 years, *n* (%)	26 (30.23)	17 (32.69)	9 (26.47)	
29–40 years, *n* (%)	40 (46.51)	27 (51.92)	13 (38.24)	
41–48 years, *n* (%)	13 (15.12)	5 (9.62)	8 (23.53)	
> 48 years, *n* (%)	7 (8.14)	3 (5.77)	4 (11.76)	
Total number of male sex partner, last 6 months, *n* (%)				0.28
0–5 male partners	7 (8.24)	5 (9.62)	2 (6.06)	
6–10 male partners	14 (16.47)	11 (21.15)	3 (9.09)	
> 10 male partners	64 (75.29)	36 (69.23)	28 (84.85)	
Receptive CAS, last 6 months, *n* (%)				0.88
0	22 (25.58)	13 (25.00)	9 (26.47)	
1 or more times	64 (74.22)	39 (75.00)	25 (73.53)	
HIV-positive male partner, last 6 months, *n* (%)				0.25
< 1 positive partner	35 (40.70)	23 (44.23)	12 (35.29)	
1 positive partner	15 (17.44)	11 (21.15)	4 (11.76)	
> 1 positive partner	36 (41.86)	18 (34.62)	18 (52.94)	
Insertive CAS, last 6 months, *n* (%)				0.06
0–4 times	67 (77.91)	44 (84.62)	23 (67.65)	
5 or times	19 (22.09)	8 (15.38)	11 (32.35)	
Syndemic-related factors				
Syndemic Count, Median (IQR)	1 (1–2)	1 (0–3)	1 (1–2)	0.84
Depressive Symptoms, last week, *n* (%)	34 (39.53)	22 (42.31)	12 (35.29)	0.51
Alcohol related problem, *n* (%)	34 (39.53)	22 (42.31)	12 (35.29)	0.51
Multiple Drug Use, last 3 months, *n* (%)	27 (31.40)	13 (25.00)	14 (41.18)	0.11
Sexual compulsivity, *n* (%)	27 (31.40)	18 (34.62)	9 (26.47)	0.43

*PrEP* pre-exposure prophylaxis, *NA*Not applicable, *CAS* condomless anal sex, *HIRI-MSM* HIV Incidence Risk Index for Men who have sex with men, *IQR* Interquartile range

^a^Chi-sq test/Exact test *p*-values for categorical variables; Wilcoxon rank-sum test *p*-values for continuous variables

^b^East Asian (10.5%), Unidentified (5.8%), Arab/Middle Eastern (4.7%), Black (3.5%), Mixed Race (2.3%), South Asian (1.2%)

The median (IQR) HIRI-MSM score of screened men was high at 26 (19–33), with the vast majority (96.5%) exceeding the recommended threshold (score ≥ 10) for defining high risk [9]. The majority reported > 10 male sex partners (75.3%) and ≥1 condomless receptive anal sex event(s) (74.2%) in the past six months. The prevalence of syndemic health problems was also high, with 39.5% meeting criteria for major depressive symptoms, 39.5% for alcohol related problems, 31.4% for multiple drug use, and 31.4% for sexual compulsivity. The median (IQR) syndemic count was 1 (1–2), with 20.9% of the sample scoring ≥3.

In the primary analyses, there was no difference in HIV risk by referral source (Table 1); the median (IQR) HIRI-MSM score was 26 (19–32.5) for self-referrals and 29 (20–34) for provider referrals (*p* = 0.28). Reporting of condomless receptive anal sex in the past 6 months was also similar between self- and provider referrals (75.0% vs. 73.5%, *p* = 0.88). However, popper use in the last three months (a component of the HIRI-MSM) was more common among provider-referrals compared to self-referrals (76.5% vs. 50.0%, *p* = 0.01). There was also a similar prevalence of all four syndemics in both groups, and an equal median (IQR) syndemic count at 1 (0–3) and 1 (1–2) among self and provider-referrals, respectively (*p* = 0.84).

After controlling for other variables in multivariable regression analyses, conclusions related to our uncontrolled analyses remained unchanged (Table 2). Being provider-referred was associated with a greater odds of popper use (vs. self-referred, aOR = 3.2, *p* = 0.03) in multivariable analysis. No other statistically significant differences were detected.

**Table 2:** Regression models exploring the association between referral source (independent variable; provider-referred vs. self-referred) and outcomes of interest (dependent variable)

	Univariable models		Multivariable models	
	MOA (95% CI)	*p*	MOA (95% CI)	*p*
Outcomes of interest (dependent variable)				
Syndemic score^a^	IRR = 0.96 (0.66, 1.38)	0.82	aIRR = 1.01 (0.72, 1.41)	0.95
Syndemic score composites^a^				
Presence of depressive symptoms, last week	OR = 0.75 (0.27, 1.98)	0.67	aOR = 0.74 (0.27, 1.98)	0.67
Presence of alcohol related problem, last year	OR = 0.75 (0.27, 1.98)	0.67	aOR = 0.91 (0.32, 2.58)	0.99
Use of multiple substance, last 3 months	OR = 2.08 (0.75, 5.87)	0.18	aOR = 2.20 (0.78, 6.44)	0.15
Presence of sexual addiction	OR = 0.68(0.23, 1.92)	0.58	aOR = 0.74 (0.24, 2.12)	0.70
HIRI-MSM score^b^	β = 2.10 (−1.77, 5.96)	0.29	aβ =2.04 (−1.84, 5.92)	0.30
HIRI-MSM score composites^b^				
Used poppers, last 3 months	OR = 3.20 (1.14, 9.78)	0.02	aOR = 3.16 (1.08, 10.12)	0.03
Used amphetamine, last 3 months	OR = 0.74 (0.15, 3.05)	0.89	aOR = 0.69 (0.14, 2.87)	0.82
Age (in years)	β = 2.74 (−1.53, 7.02)	0.21	aβ = 2.66 (−1.39, 6.72)	0.20
Total number of male sex partners, last 6 months				
≤ 10 male partners	Ref.		Ref.	
> 10 male partners	OR = 2.46 (0.74, 9.67)	0.17	aOR = 2.35 (0.72, 9.15)	0.19
Number of receptive CAS male partners, last 6 months				
0 times	Ref		Ref	
1 or more times	OR = 0.93 (0.31, 2.89)	0.99	aOR = 0.93 (0.31, 2.81)	0.99
Number of HIV-positive male sex partners, last 6 months				
< 1 positive partner	Ref		Ref	
≥ 1 positive partner	OR = 1.45(0.55, 3.94)	0.55	aOR = 1.43 (0.55, 3.87)	0.56
Number of insertive CAS HIV-positive male partners, last 6 months				
0–4 times	Ref		Ref	
5 or more times	OR = 2.59 (0.82, 8.61)	0.11	aOR = 2.82 (0.88, 9.55)	0.09

Measure of association (MOA) in each row is derived from a separate regression model and refers to the association between the referral source (independent variable; self-referred = referent category) and outcome of interest (independent variable). An OR or IRR of greater than 1 (or a positive β value) indicates that being provider-referred was positively associated with the outcome of interest. For example, being provider-referred was associated with a lower odds of depressive symptoms (OR = 0.75), but the finding was not statistically significant (95% CI 0.27–1.98). MOA: Measure of Association; CI: Confidence Interval; β: beta coefficient from linear regression; aβ: adjusted beta coefficient from linear regression; IRR: incident rate ratio from Poisson regression; aIRR: adjusted incident rate ratio from Poisson regression; OR: odds ratio from logistic regression; aOR: adjusted odds ratio from logistic regression; HIRI-MSM: HIV Incidence Risk Index for Men who have Sex with Men; CAS: Condomless Anal Sex; CES-D: Center for Epidemiologic Studies Depression Scale; AUDIT: Alcohol Use Disorder Identification Test; SCS: Sexual Compulsivity Scale. ^a^Multivariables models are controlled for age, race/ethnicity, and education; ^b^Multivariables models are controlled for race/ethnicity, and education

Finally, in post-hoc analyses exploring whether the number of mental health conditions (syndemic score) modified the relationship between HIV risk and referral source, we found that the interaction term between HIRI-MSM score and syndemic score was not significant (*p* = 0.75).

## Discussion

Uncertainty exists with regards to the role of CBOs and non-clinical services and providers in the implementation of “biomedical” interventions such as PrEP [28, 29], but our findings suggest that online strategies and CBOs working in gay men’s health can play important roles in connecting individuals at high HIV risk to PrEP services. The rate of gbMSM referrals to our study was high, and similar to that observed in open label PrEP studies in the United States [2, 30]. Contrary to our hypothesis, however, the level of HIV risk and burden of syndemic health problems were similarly high among gbMSM who self-referred to this pilot PrEP demonstration project, compared to those who were provider-referred.

Men screened for participation in our PrEP demonstration project were at high risk of HIV infection. The median (IQR) HIRI-MSM score of 26 (19–33) among screened gbMSM was much higher than in a 2014–2015 sample of over 400 gay men testing for HIV at a busy sexual health clinic in downtown Toronto (median = 15, IQR = 8–19) [7]. This finding is consistent with other demonstration projects showing that PrEP attracts gay men at highest risk of HIV infection [31, 32]. In the original cohorts used to derive the HIRI-MSM, only 8–9% scored 26 or greater, and this threshold was associated with a specificity of 92–93% for predicting HIV infection in the next 6 months [9]. Further, a recent modeling study identified a HIRI-MSM score of 25 or more as a cost-effective threshold for targeting PrEP to MSM. While this modeling study is subject to the same limitations inherent to the HIRI-MSM tool (discussed further below), it suggests PrEP in Toronto may be cost-effective if the HIV risk profile in future users is as high as in our sample [33].

Screened men also had a high burden of syndemics. Approximately one-third were identified as having depressive symptoms, an alcohol-related problem, multiple drug use, or sexual compulsivity and one-fifth had three to four of these problems. This high prevalence is consistent with data showing that syndemic health problems concentrate in urban gbMSM and synergize to produce high HIV risk [15, 16, 34], and also highlights the potential to use PrEP programs as a gateway to other health services [35]. Another study from our group identified a similarly high burden of syndemic problems among patients accessing nPEP [18], and we have elsewhere argued for the routine implementation of screening strategies for such syndemics in all PrEP programs [17]. Multiple drug use in our study was similar to the baseline prevalence among gbMSM enrolled in the US PrEP Demonstration Project, where 20.1% reported use of 3 or more of poppers, ketamine, ecstasy, gamma-hydroxybutyrate, cocaine, methamphetamine, or erectile dysfunction drugs in the past 3 months [30]. Interventions (eg. peer navigators [36]) to link PrEP users to relevant health services (e.g. psychosocial, medical and mental), along with data evaluating the impact of such services on PrEP-related outcomes, are urgently needed.

To our knowledge, only one other PrEP demonstration project has compared screened gbMSM by referral source. In that study, PrEP was integrated into STD clinics in San Francisco and Miami, as well as a community health center in Washington, D.C., and self-referred individuals exhibited greater HIV risk behaviour and greater perceived HIV risk than those who were clinic-referred [37]. Further, self-referred men were also more likely to initiate PrEP [37] and, among those who started PrEP, there was a trend towards better adherence (*p* = 0.07) and retention (*p* = 0.08) among self-referred men [30]. There may be several reasons for the discrepancy between this study’s findings and our own. First, in the US-based study, participants initiated PrEP in the same clinic where the client was identified, while in our study providers referred participants to an external study site. That our provider-referred participants had to complete the extra step of attending the study site likely meant they were more highly motivated to seek PrEP (and thus more similar to self-referrals), potentially explaining the lack of difference by referral type in our study. Second, the “self-referral” category in the US-based study included individuals who were referred to the PrEP clinic by their primary care providers; this group would have been classified as provider-referred in our study. Third, in our study, self-referrals primarily came from Grindr and provider-referrals from a popular community-based STI clinic in Toronto, both of which have been previously shown to attract high risk gay men [7, 38]. Therefore, it may not be surprising that risk profiles were similarly high by referral group. However, not all of those frequenting these services are at high risk, and it is reassuring that data from both our study and the US-based demo project suggest that many gbMSM recognize their elevated HIV risk and potential need for PrEP, and that promoting self-referrals is an important strategy for identifying appropriate PrEP candidates. Further, our results suggest that Grindr and community-based STI clinics can play important roles in promoting future PrEP uptake.

Many self-referrals in our study were referred by friends or through word of mouth, suggesting an important role for social networks in improving awareness and uptake of biomedical HIV technologies [39, 40]. Marketing campaigns designed to promote diffusion of information through social networks may facilitate more widespread PrEP self-referrals among individuals at high risk [41]. Importantly, interventions to promote self-referrals will need to simultaneously address the lack of knowledgeable providers to which people can refer themselves. To overcome this barrier, our team is currently evaluating a strategy in which patients themselves link their providers with accredited continuing medical education resources on PrEP [42].

Provider-referrals will also remain important to promoting PrEP uptake, particularly for objectively high risk gbMSM who underestimate their HIV risk [7], though such men may be difficult to reach if they are not engaged in care or “out” to their provider. Although we found no significant difference in perceived HIV risk by referral type, our study was underpowered for this comparison. Expanding continuing medical education on PrEP and the promotion of clear clinical indications for its use could increase provider-initiated PrEP in future.

Strengths of our study include being Canada’s first PrEP demonstration project and our use of validated scales and concealment of eligibility criteria to minimize reporting bias during participant screening. Our study also has limitations that warrant consideration. First, our modest sample size may have limited our ability to detect small differences between referral sources. Second, because PREPARATORY-5 was the first opportunity within Toronto’s gay community to access PrEP at no cost, those seeking study participation may have been “early adopters” and different from the broader gay community. However, the impact of such differences on our primary research questions regarding referral sources is unknown. Further, our sample was mostly White, college educated, and previously aware of PrEP – potentially limiting generalizability of our results. Differences between provider- and self-referrals may become more apparent in more diverse populations, as previous studies have shown that ethnic minorities and individuals with less education are less likely to self-refer [37] and that providers may have racial and other biases in prescribing PrEP [43]. Third, although our findings suggest that providers can accurately identify HIV risk, it was not feasible to determine whether formal risk assessments were actually conducted for provider-referred participants.

Finally, we used the HIRI-MSM tool to measure HIV risk, which has several inherent limitations that our team has previously described [44]. In particular, this tool has not been validated in our setting and was derived from US-based cohorts of gay men conducted over a decade ago [9]. As such, the index does not include several HIV risk factors that have become important in the modern context, including viral load and PrEP use. Further, the tool does not include upstream HIV risk factors, including mental health problems, and may not reflect an individual’s HIV risk at the time of measurement or within the short-term future, as the HIRI-MSM is retrospective in nature and HIV risk is dynamic. Regardless, there were no significant differences by referral source in almost any components of the HIRI-MSMrisk score (many of which are common proxies for sexual HIV risk in other studies of gay men) or mental health problems which are commonly linked to higher HIV risk.

## Conclusions

The high rate of PrEP referrals and prevalence of syndemic problems (which are closely associated with HIV risk) in our study population highlight unmet health needs in Toronto’s gay community. Our study suggests that strategies to promote both provider and self-referrals for PrEP are needed to reach at-risk individuals who could benefit most.

## Abbreviations

AUDIT: Alcohol Use Disorders Identification Test
CBO: Community-based organization
CES-D: Center for Epidemiological Studies - Depression
gbMSM: gay, bisexual and other men who have sex with men
HIRI-MSM: HIV Incidence Risk Index for Men who have Sex with Men
IQR: Interquartile range
nPEP: non-occupational post-exposure prophylaxis
PrEP: Pre-exposure prophylaxis
SCS: Sexual Compulsivity Scale
